# Novel Siloxane-Modified Epoxy Resins as Promising Encapsulant for LEDs

**DOI:** 10.3390/polym12010021

**Published:** 2019-12-20

**Authors:** Chih-Hao Lin, Wha-Tzong Whang, Chun-Hua Chen, Shu-Chen Huang, Kai-Chi Chen

**Affiliations:** 1 Department of Materials Science and Engineering, National Chiao Tung University, 1001 University Rd., Hsinchu 300, Taiwan; howardlin@itri.org.tw (C.-H.L.); wtwhang@mail.nctu.edu.tw (W.-T.W.); 2 Material and Chemical Research Laboratories, Industrial Technology Research Institute, 195, Sec. 4, Chung Hsing Rd., Chutung 31040, HsinChu, Taiwan; dogcoach432@gmail.com (S.-C.H.); vickychen@itri.org.tw (K.-C.C.)

**Keywords:** siloxane-modified epoxy, crosslinking density, sulfurization resistance, surface mounted device LEDs, encapsulant

## Abstract

This study investigated a new category of transparent encapsulant materials for light-emitting diodes (LEDs). It comprised a phenyl group that contained siloxane-modified epoxy (SEP-Ph) hybridized with a cyclic tetrafunctional siloxane-modified epoxy (SEP-D4) with methylhexahydrophthalic anhydride (MHHPA) as a curing agent. The SEP-Ph/SEP-D4 = 0.5/0.5 (sample 3) and SEP-D4 (sample 4) could provide notably high optical transmittance (over 90% in the visible region), high-temperature discoloration resistance, low stress, and more crucially, noteworthy sulfurization resistance. The lumen flux retention of the SEP encapsulated surface mounted device LEDs remained between approximately 97% and 99% after a sulfurization test for 240 h. The obtained comprehensive optical, mechanical, and sulfurization resistance proved the validity and uniqueness of the present design concept with complementary physical and chemical characteristics.

## 1. Introduction

Light-emitting diodes (LEDs) have become an essential light source because of their compact size, high efficiency, high brightness, low power consumption, and long operating lifetime. The performance of LEDs is greatly affected by the properties of their encapsulation materials, such as high optical transmittance, thermal- and photostability, low stress, and gas permeation resistance [1,2,3,4,5,6]. Transparent encapsulation materials are used to protect LED chips and can generally be divided into two main categories: epoxy resins [7,8] and silicone resins [9].

Transparent epoxy resins cured with anhydrides possess good optical clarity, strong adhesion, and high mechanical strength; however, their poor thermal- and photostability, as well as high thermal stress result in discoloration of and catastrophic damage to LED devices, which restricts their application. By contrast, silicone resins have excellent thermal- and photostability, and are thus usually applied in mid- to high-power LED packaging; however, their weak adhesion and high gas permeability, mainly originating from their highly flexible siloxane backbone, which allows for gas diffusion [10,11], are crucial concerns for some applications.

A series of siloxane-modified epoxy resins have been extensively investigated with the aim of combining the merits of epoxy and silicone resins [12,13,14,15,16,17,18]. Morita et al. synthesized an epoxy siloxane monomer with a cycloaliphatic epoxy group at the terminal of the siloxane chain and demonstrated improved thermal- and photostability through curing with appropriate quantities of anhydride hardener and accelerator [14,15]. Huang et al. developed a series of cyclic silicone epoxies with various numbers of epoxy groups. By curing with aluminum acetylacetonate (Al(acac)_3_) and diphenylsilanediol (Ph_2_Si(OH)_2_), enhanced ultraviolet stability was achieved compared with an anhydride-cured cycloaliphatic epoxy [16]. Hue et al. prepared a transparent composition using the diglycidyl ether of bisphenol A and a phenylmethylsiloxane-modified epoxy (PMSE) hybrid resin. Curing with methylhexahydrophthalic anhydride (MHHPA) revealed that the addition of an appropriate amount of PMSE could effectively improve high-temperature thermal stability, dynamic mechanical stability, and performance of LEDs [18].

Currently, surface mounted device LEDs (SMD LEDs) are advancing and their packages are becoming smaller and thinner. This allows harmful contaminants in the atmosphere such as hydrogen sulfide (H_2_S) to easily permeate an LED’s package through its transparent encapsulation material. In this case, the bottom silver electrodes would darken because of the formation of silver sulfide, which would be accompanied by a significant decrease in the LED’s light output. Although transparent epoxy-based encapsulants exhibit suitable gas barrier properties, the high thermal stress of epoxy resins that is generated during the soldering or temperature-cycling process would cause catastrophic damage. By contrast, silicone materials possess relatively low thermal stress compared with conventional epoxy resins, but their higher gas permeation properties would pose a problem for thin SMD LED packages, especially in outdoor applications.

Many research studies have been conducted to enhance the gas barrier properties of transparent polymers, one of which added nanoscale lamellar fillers with a large aspect ratio, such as clay or graphene, into the polymer matrix [19,20]. The addition of clay can alter and extend the gas molecule diffusion pathway; furthermore, the incorporation of graphene can effectively prevent gas molecules from permeating into the polymer matrix; however, the aggregation of nanoscale fillers can result in opacity, significantly reducing the optical transmittance of the polymer matrix. Moreover, the addition of clay can accelerate its rate of photooxidation [20]. The second option is to employ vapor deposition or coat a dense inorganic or organic material (e.g., SiOx, Al_2_O_3_, or organic polysilazane) onto the surface of the polymer matrix. However, this approach requires special facilities (e.g., CVD and vacuum conditions) and complicated processes [21,22,23].

In this study, we designed a promising transparent material with compromised properties, namely high optical transmittance, high thermal stability, low modulus, and high sulfurization resistance, through blending the phenyl group-containing siloxane-modified epoxy (SEP-Ph) and cyclic tetrafunctional siloxane-modified epoxy (SEP-D4). The structure of SEP-Ph has multiple epoxy groups and flexible siloxane segments, which have the benefit of reducing the modulus without sacrificing the glass transition temperature (*T*_g_). Moreover, the rigid structure of SEP-D4 with tetra epoxy groups could contribute thermal dimensional stability and high crosslinking density, and, more crucially, high sulfurization resistance should be obtained for transparent encapsulants.

## 2. Materials and Methods

### 2.1. Materials

**Figure 1:**
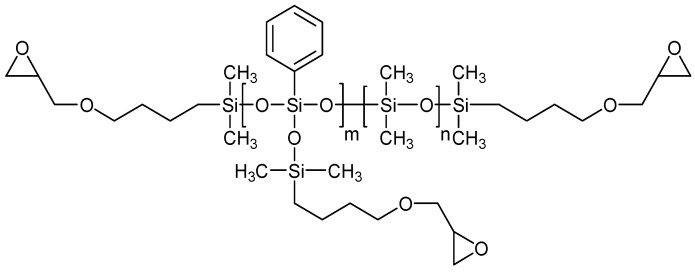
Structure of phenyl group-containing multi-functional siloxane-modified epoxy SEP-Ph.

Hydride-terminated 1,3,5,7-tetramethylcyclotetrasiloxane and tris(dibutylsulfide)rhodium trichloride were obtained from Gelest (Morrisville, PA, USA). The phenyl group-containing siloxane-modified epoxy (SEP-Ph, EEW = 670; see Figure 1) was provided by Grandtek Co. Ltd. (Hsinchu, Taiwan). Furthermore, 4-vinyl-1-cyclohexene-1,2-epoxy was purchased from Aldrich (St. Louis, MO, USA); N,N-dioctadecylmethylamine was supplied by Fluka (Tokyo, Japan); charcoal active powder was obtained from SHOWA (Saitama, Japan); methylhexahydrophthalic anhydride (MHHPA) was supplied by New Japan Chemical Co., Ltd. (Osaka, Japan); and the special amine curing accelerator U-CAT 18X was purchased from San-Apro (Kyoto, Japan).

### 2.2. Synthesis of 1,3,5,7-Tetramethyl-1,3,5,7-Tetra[(3,4-Epoxycyclohexyl)Ethyl]-Cyclotetrasiloxane (SEP-D4)

**Scheme 1:**
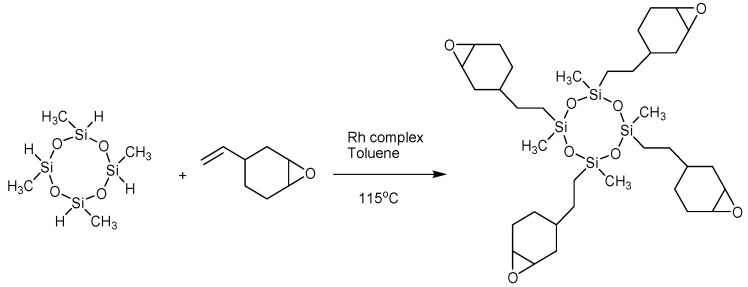
Synthesis of 1,3,5,7-tetramethyl-1,3,5,7-tetra[(3,4-epoxycyclohexyl)ethyl]-cyclotetrasiloxane.

The synthesis of 1,3,5,7-tetramethyl-1,3,5,7-tetra[(3,4-epoxycyclohexyl)ethyl]-cyclotetrasiloxane (SEP-D4) was conducted according to the hydrosilylation reaction [24,25]. Scheme 1 illustrates the synthesis route. First, 50 g of 1,3,5,7-tetramethylcyclotetrasiloxane (0.21 mol) and 150 mL of toluene were added to a 1-L, three-necked, round-bottomed flask filled with high-purity nitrogen. Then, the mixture was heated to 100 °C and stirred for 20 min. Next, 2.55 g of tris(dibutylsulfide)rhodium trichloride diluted solution (0.8 g of tris(dibutylsulfide)rhodium trichloride dissolved in 80 g of toluene) was added to the mixture before it was stirred for another 20 min at 100 °C. Subsequently, 115 g of 4-vinyl-1-cyclohexene-1,2-epoxy (0.84 mol) was added dropwise into the reaction mixture; next, the reaction temperature was raised to 115 °C and the mixture was stirred for 72 h. Fourier-transform infrared spectroscopy was used to monitor the reaction completely through the disappearance of 2150 cm^−1^ for Si–H bonds. The hydrosilylation reaction was terminated by cooling the reaction mixture to room temperature and adding active carbon to deactivate the catalyst. After filtering the active carbon in the reaction mixture, the unreacted materials and solvent were removed through rotary evaporation at 80 °C to give the colorless transparent liquid products of 1,3,5,7-tetramethyl-1,3,5,7-tetra[(3,4-epoxycyclohexyl)ethyl]-cyclotetrasiloxane, ^1^H NMR: 0.04 ppm (s, CH_3_–Si), 0.48 ppm (m, –CH_2_–Si), 0.17–2.18 ppm (m, cyclohexyl group), 3.12 ppm (m, epoxy group), and EEW = 184.

### 2.3. Perpetration of Transparent SEP-Ph/SEP-D4/MHHPA Compositions

Table 1 lists the mixing ratios by the equivalent weight of SEP-Ph/SEP-D4/MHHPA compositions. U-CAT 18X was used as a reaction accelerator (0.3 per hundred resin (phr) based on the total amount of SEP-Ph and SEP-D4) and mixed with MHHPA by stirring vigorously at room temperature until homogeneity was achieved. Then, SEP-Ph, SEP-D4, and the mixtures of SEP-Ph/SEP-D4 in different equivalent weight ratios were added to the abovementioned solution as well as stirred until homogeneous to form the final compositions. These compositions were then poured into a glass mold, degassed in a vacuum oven, and finally cured at 130 °C for 1 h followed by 150 °C for another 2 h.

### 2.4. Instrumentation

^1^H-NMR analysis was performed on a NMR spectrometer (Varian 500 MHz; Agilent, Santa Clara, CA, USA) with CDCl_3_ as the solvent and TMS as a reference. Differential scanning calorimetry (DSC Q10; TA Instrument, New Castle, DE, USA) analysis was conducted under a nitrogen atmosphere. Approximately 10 mg of the testing sample was sealed in an aluminum pan. The samples were heated from 40 to 300 °C at a rate of 10 °C/min. The onset temperature, peak temperature, and enthalpy of the exothermic peak of the curing compositions were recorded. Modulated DSC (mDSC Q2000; TA Instrument, New Castle, DE, USA) analysis was conducted under a nitrogen atmosphere. Approximately 10 mg of the testing sample was sealed in an aluminum pan. The sample was equilibrated at −60 °C, modulated at ±1.00 °C every 60 s as well as isothermally for 5 min, and then heated to 250 °C at a heating rate of 5 °C/min. The glass transition temperature (*T*_g_) was determined by the inflection point of the reverse heat flow curve. Thermal degradation properties were measured using a TGA (TGA 7; Perkin Elmer, Waltham, MA, USA) from 30 °C to 800 °C at a heating rate of 10 °C/min. Dynamic mechanical analysis (DMA Q800; TA Instrument, New Castle, DE, USA) was performed at a heating rate of 5 °C/min from −100 to 280 °C at a fixed frequency of 1 Hz in single cantilever mode. The dimensions of the test specimen were 17.5 mm (*L*) × 12.8 mm (*W*) × 3.2 mm (*T*). Optical transmittance from 350 to 800 nm was measured using UV/Vis spectrometer (Lambda 950; Perkin Elmer, Waltham, MA, USA) and the sample thickness was 2 mm. Thermal mechanical analysis (TMA Q400; TA Instrument, New Castle, DE, USA) was conducted at a heating rate of 10 °C/min from −50 °C to 250 °C. Optical properties of the LED were measured using an integrating sphere (ISP-250; Instrument Systems, Munich, Germany) with a driving current of 150 mA.

### 2.5. Encapsulation of SMD LEDs

In this study, EMC3030 (Sander Electronics, Taipei, Taiwan) SMD LEDs bonded with a 350 mA blue LED chip were used for the optical-device testing vehicle. Before encapsulation, the lead frame was baked at 140 °C for 2 h to remove moisture, followed by the compositions being dispensed into the cavity of the EMC3030 SMD LED. The samples in this study were cured in a high-temperature oven with curing profiles of 130 °C for 1 h and 150 °C for 2 h. The commercial silicone was cured with curing profiles of 100 °C for 1 h and 150 °C for 5 h. After encapsulation, the optical property of luminous flux was measured using the integrating sphere.

### 2.6. Sulfurization Test of the Encapsulated SMD LEDs

This study performed a sulfurization test on the SMD LEDs encapsulated by the transparent siloxane-modified epoxy compositions and commercial silicone to evaluate their sulfurization resistance. The encapsulated LEDs were attached to the wall side of a 100-mL glass sample flask; 0.2 g of sulfur powder was placed in the flask and closed, and then the flask was placed in a high-temperature oven at 70 °C. Changes in color of the SMD LEDs’ silver electrodes were observed during the sulfurization test period. Moreover, the light-output performance of the LED was recorded using the integrating sphere.

## 3. Results and Discussion

### 3.1. Curing Behavior and Miscibility of the Transparent Siloxane-Modified Epoxy Compositions

**Figure 2:**
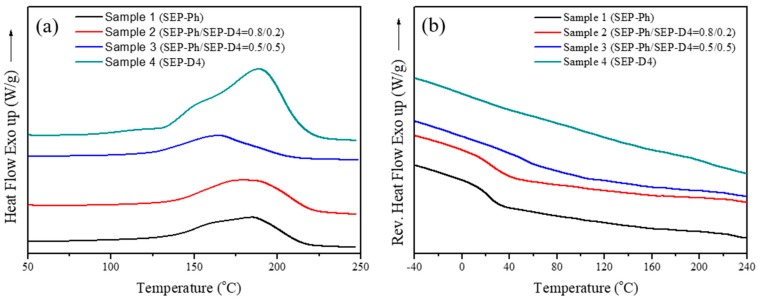
(**a**) Exothermic curves and (**b**) reverse heat flow curves of the transparent siloxane-modified epoxies.

The DSC analysis (see Figure 2a) revealed broad exothermic peaks for the four transparent siloxane-modified epoxy compositions. The exothermic onset temperatures were 132, 133, 122, and 128 °C, and the peak temperatures were 185, 184, 165, and 190 °C for samples 1, 2, 3, and 4, respectively. The curing behavior of samples 1 and 2 were similar. It might be due to the SEP-Ph/SEP-D4 were in a suitable balance of epoxy equivalent ratio of 0.5/0.5, both the exothermic onset and peak temperatures decreased significantly, indicating superior reactivity compared with the others. Moreover, the curing heats of samples 1, 2, 3, and 4 were 130, 146, 197, and 311 J/g, respectively. The curing heat increased with the decreasing epoxy equivalent weight. In siloxane-modified epoxy, the larger epoxy equivalent indicated a greater amount of the dimethyl siloxane unit, which decreased the curing heat. The glass transition temperature (*T*_g_) was determined using the inflection point of the reverse heat flow curve of mDSC, as displayed in Figure 2b. Samples 2 and 3 exhibited single inflection points, and the single *T*_g_ was evidence of the blending of SEP-Ph and SEP-D4 being miscible with no obvious phase separation. By contrast, the reverse heat flow curve of SEP-D4 did not exhibit any significant inflection, which was probably because of the highly rigid network restricting the molecular mobility.

### 3.2. Optical Properties and Thermal Discoloration Resistance of the Transparent Siloxane-Modified Epoxy Compositions

**Figure 3:**
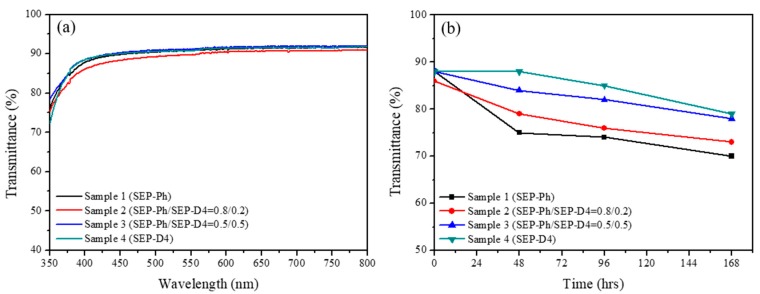
(**a**) UV/Vis spectra of the transparent siloxane-modified epoxies before thermal aging at 150 °C, and (**b**) variation in transmittance at 400 nm as a function of time after thermal aging at 150 °C.

High optical transmittance and thermal discoloration resistance are basic requirements for transparent materials in optical-device applications. Figure 3a illustrates the optical transmittance of four cured transparent siloxane-modified epoxy compositions. The initial optical transmittance from 450 to 800 nm was over 88% with a sample thickness of 2 mm, which suggested that materials with high optical transparency are required for LED encapsulation. Subsequently, thermal discoloration was evaluated by estimating the reduction in transmittance at 400 nm, which was caused by the significant decline in transmittance in short-wavelength regions as discoloration occurred. Figure 3b illustrates the variation in transmittance at 400 nm after 150 °C thermal aging of the transparent siloxane-modified epoxy compositions for 48, 96, and 168 hours. The initial transmittance values at 400 nm of samples 1–4 were 88%, 86%, 88%, and 88%, and decreased to 70%, 73%, 78%, and 79% after thermal aging at 150 °C for 168 hours. Significant transmittance decay was observed at 400 nm in samples 1 and 2 (18% and 13%, respectively); by contrast, samples 3 and 4 exhibited relatively low transmittance decay at 400 nm of 10% and 9%, respectively. As a result, samples 3 and 4 exhibited similar thermal discoloration resistance, which was superior to that of samples 1 and 2. Furthermore, the incorporation of SEP-D4 effectively enhanced the thermal discoloration resistance of SEP-Ph. The thermal discoloration resistance of the transparent siloxane-modified epoxy system in this study was similar to that in a previous study [26].

### 3.3. Thermal Decomposition and Mechanical Properties of the Transparent Siloxane-Modified Epoxy Compositions

**Figure 4:**
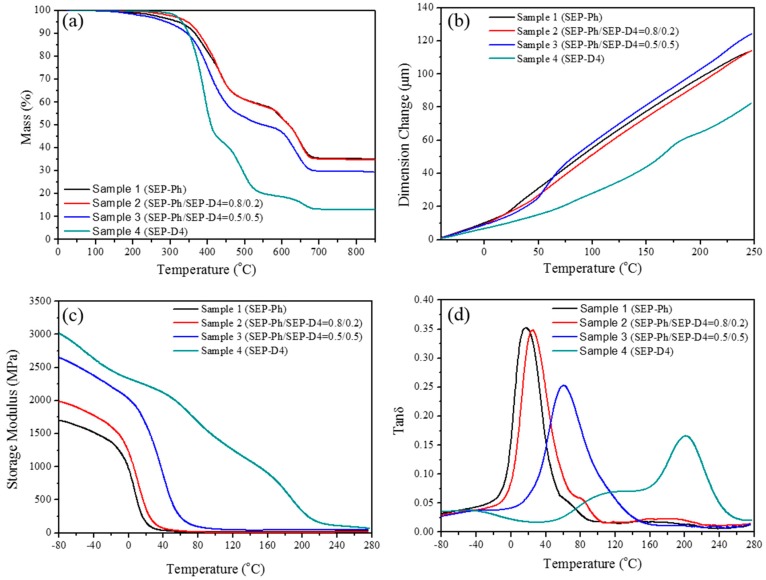
(**a**) Thermogravimetric analsis (TGA) curves and (**b**) Thermal mechanical analysis (TMA) curves of the transparent siloxane-modified epoxies. (**c**) Storage modulus and (**d**) tanδ curves of the cured transparent siloxane-modified epoxies.

The thermal decomposition of samples 1, 2, 3, and 4 were studied using thermogravimetric analysis (TGA). Figure 4a shows the weight loss as a function of temperature. The onset decomposition temperatures were 376, 367, 353, and 348 °C. The higher onset decomposition temperatures of samples 1–4 can ensure that no decomposition occurs under high-temperature processing conditions (e.g., during reflow process) in LED manufacturing.

In addition, the thermal dimensional stability and mechanical properties of transparent siloxane-modified epoxy compositions are critical for their application [27,28]. Therefore, the thermal dimensional stability of the compositions in this study was studied using thermal mechanical analysis (TMA). Figure 4b presents dimension changes as a function of the temperature curve; the coefficients of thermal expansion (CTE) below *T*_g_ (α1) of samples 1–4 were 101, 94, 89, and 64 ppm/°C, respectively. Sample 1 exhibited the highest α1 because of the flexible siloxane backbone of SEP-Ph, and it was reduced through incorporating a rigid structure of SEP-D4. A reason for this could be that introducing this rigid structure helped to restrict the segment motion of the transparent siloxane-modified epoxy, thereby lowing α1. This study noticed that the α1 values of samples 1–4 were lower than that of the epoxy siloxane polymer that was prepared and investigated by Morita et al. [15]. Accordingly, superior thermal dimensional stability would be obtained.

Dynamic mechanical properties were evaluated using DMA analysis, which was conducted from −100 to 280 °C. The storage modulus is the elastic component of a material and is related to its stiffness. Figure 4c presents the storage modulus curve as a function of temperature. The storage modulus values of samples 1–4 at 25 °C was 97, 230, 1491, and 2210 Mpa, respectively, which were lower than those of conventional cycloaliphatic epoxy and the silicone-epoxy resins studied by Yang et al. [29]. The storage modulus of samples 1–3 demonstrated an increasing trend with increasing quantities of SEP-D4, whereas sample 4 exhibited the highest storage modulus. This phenomenon could have originated from the rigid structure of SEP-D4 reinforcing and increasing the crosslinking density of transparent siloxane-modified epoxy systems.

The crosslinking densities of samples 1–4 were studied according to the storage modulus in a rubbery state. In general, a polymer material with a high crosslinking density usually exhibits a high storage modulus in the rubbery region. The crosslinking densities of samples 1–4 were calculated using the following equation [30,31] and the results are summarized in Table 2:

*ρ* = *E*′/3*RT*

where *ρ* is the crosslinking density, *E*′ is the storage modulus at temperature *T* (*T* = *T*_g_ + 50 K and represents absolute temperature), and *R* is the gas constant (8.314 J·K^−1^·mol^−1^).

The results indicated that as the quantity of SEP-D4 increased, the crosslinking density also increased, because of the increasing crosslinking point. Sample 4 had the highest crosslinking density among the prepared transparent siloxane-modified epoxy materials. A high crosslinking density is believed to be beneficial for reducing the free volume and network flexibility of siloxane-modified epoxy materials, and thus improving their sulfurization resistance [32,33,34].

Tanδ, which is defined as the ratio of loss modulus to storage modulus, is usually used to estimate the damping property of a material. Damping is the dissipation of energy in a material under cyclic loading. The glass transition temperature can also be determined using the peak value of tanδ (see Figure 4d). In this study, the glass transition temperature of SEP-D4 was much higher than those of the transparent siloxane-modified epoxy compositions with different amounts of SEP-Ph. The *T*_g_ values of samples 1–4 were 17, 25, 61, and 200 °C, and their peak tanδ values were 0.352, 0.348, 0.253, and 0.183, respectively. According to these results, this study concluded that increasing the amount of SEP-D4 results in a higher glass transition temperature, lower tanδ, and lower network flexibility, thereby, decreasing material’s energy dissipation ability.

### 3.4. Sulfurization Resistance of the Transparent Siloxane-Modified Epoxy Compositions

**Figure 5:**
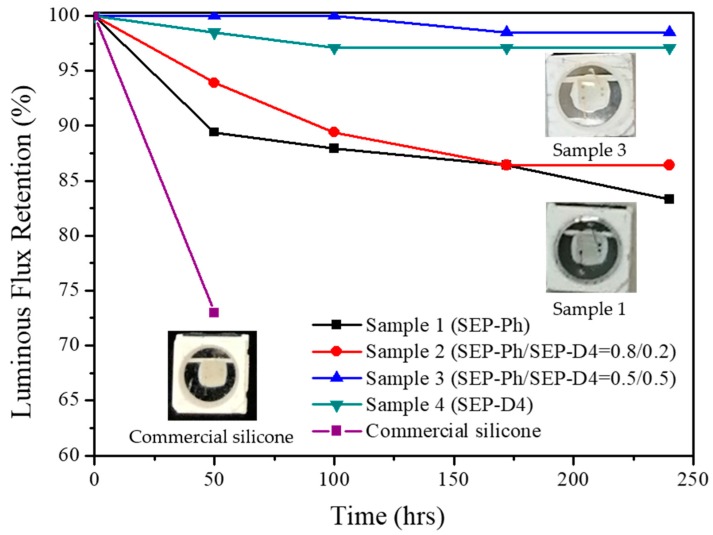
Luminous flux retention curve as a function of the sulfur test time of the prepared transparent siloxane-modified epoxies and commercial silicone.

The sulfurization resistance of the four transparent siloxane-modified epoxy compositions was evaluated with a sulfurization test of the encapsulated EMC3030 SMD LEDs at 70 °C. The results were compared with those of a commercial silicone encapsulant and are displayed in Figure 5 and Table 3. The initial average luminous flux values of the EMC3030 SMD LEDs encapsulated by samples 1–4 and commercial silicone were 6.6, 6.6, 6.6, 6.8, and 6.7 lm, respectively. The commercial silicone exhibited poorer sulfurization resistance than the transparent siloxane-modified epoxy compositions; the average luminous flux retention of the EMC3030 SMD LEDs encapsulated by commercial silicone exhibited an obvious decrease to 73.1% after the sulfurization test for 48 h, because the silver electrode of the LED was corroded by the permeating sulfur molecules and its surface darkened. Consequently, the partial light output from the LED chip was absorbed, resulting in luminous flux decay. By contrast, the sulfurization resistance of samples 1 and 2 was superior to that of the commercial silicone; furthermore, the average luminous flux retentions of samples 1 and 2 were 83.3% and 86.4%, respectively, as the testing time was extended to 240 h. Furthermore, the EMC3030 SMD LEDs encapsulated by samples 3 and 4 exhibited superior sulfurization resistance compared with samples 1 and 2 and the commercial silicone. Moreover, the average luminous flux retentions of samples 3 and 4 were 98.5% and 97.1%, respectively, after the sulfurization test for 240 h, and the silver electrode remained in its initial state. These results indicated that the sulfurization resistance of the prepared transparent siloxane-modified epoxy compositions was greatly improved through increasing the quantity of SEP-D4 in the compositions to an appropriate level.

**Figure 6:**
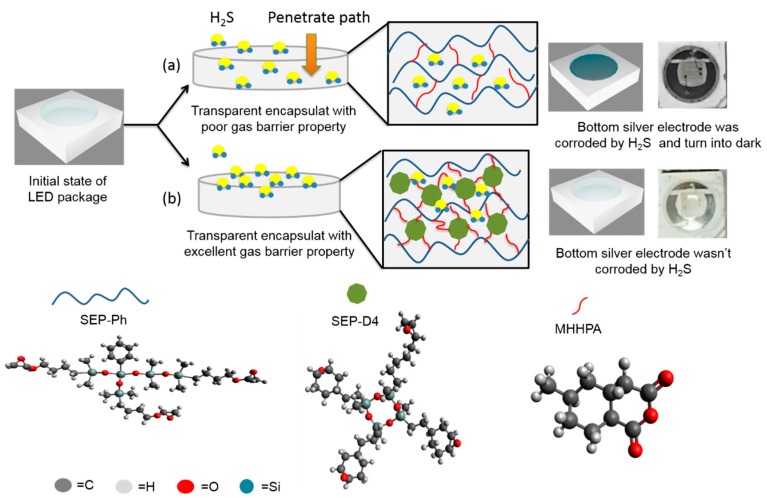
(**a**) Hydrogen sulfide (H_2_S) could not only easily permeate into the phenyl group-containing siloxane-modified epoxy (SEP-Ph), but reacted with the bottom silver electrodes, which lead to the significant decay of the LED’s light-output performance. (**b**) Schematic of the proposed key strategy for enhancing the sulfurization resistance of SEP-Ph through blending cyclic tetrafunctional siloxane-modified epoxy (SEP-D4), thereby increasing the crosslinking density of the siloxane-modified epoxy material.

In addition, the sulfurization resistance behaviors of the EMC3030 SMD LEDs encapsulated by samples 1–4 were found to be correlated with crosslinking density, and are illustrated in Figure 6. Samples 1 and 2 had relatively low crosslinking densities, which could reasonably explain their relatively low sulfurization resistance; by contrast, samples 3 and 4 possessed relatively high crosslinking densities, which demonstrated their promising sulfurization resistance.

Adding appropriate amounts of SEP-D4 into the SEP-Ph composition led to increased crosslinking density and reduced network flexibility. Therefore, acceptable sulfurization resistance could be obtained. Notably, after the LEDs were exposed to sulfur gas for 24 h, those encapsulated with samples 3 and 4 exhibited higher flux retention than that encapsulated with only trifluoropropyl phenyl silicone rubber or even with an additional protective polysilazane top layer [21]. The much longer duration time of approximately 240 h provided strong evidence of the durability of the present siloxane-modified epoxy recipe.

## 4. Conclusions

In summary, this study investigated a siloxane-modified epoxy material with high transparency, good thermal discoloration resistance, low thermomechanical stress, and promising sulfurization resistance through incorporating SEP-D4 into SEP-Ph and an MHHPA curing agent composition. SEP-D4 exhibited a high capacity with SEP-Ph and MHHPA in various equivalent ratios. The results indicated that both samples 3 and 4 had high transmittance (approx. 90%) in the visible light region, high thermal discoloration resistance (transmittance at 400 nm decayed by 10% and 9% after thermal aging at 150 °C for 168 h). Furthermore, the lumen flux retention of the LED encapsulated by these compositions approached 97%–99% after a sulfurization test for 240 h. The comprehensive properties of the transparent siloxane-modified epoxies of samples 3 and 4 demonstrated promising properties for LED package applications.

## Figures and Tables

**Table 1:** Transparent siloxane-modified epoxy compositions.

Sample ID	Sample 1	Sample 2	Sample 3	Sample 4
Compound	SEP-Ph	SEP-Ph/SEP-D4 = 0.8/0.2	SEP-Ph/SEP-D4 = 0.5/0.5	SEP-D4
SEP-Ph	1	0.8	0.5	0
SEP-D4	0	0.2	0.5	1
MHHPA	1	1	1	1

**Table 2:** Crosslinking densities of cured transparent siloxane-modified epoxy compositions.

Sample	*T* (K)	*E*′ @*T* (MPa)	*ρ*(10^−3^ mol/cm^3^)
**Sample 1**	340.15	15.52	1.83
**Sample 2**	350.15	18.22	2.00
**Sample 3**	384.15	49.11	5.13
**Sample 4**	523.15	97.76	7.49

**Table 3:** Optical properties of EMC3030 surface mounted device light-emitting diodes (SMD LEDs) before and after the sulfurization test.

Sample	Luminous Flux Before Sulfur Test (lm)	Luminous Flux After Sulfur Test for 240 h (lm)	Luminous Flux Retention Percentage (%)
**Sample 1**	6.6	5.5	83.3
**Sample 2**	6.6	5.7	86.4
**Sample 3**	6.6	6.5	98.5
**Sample 4**	6.8	6.6	97.1
**Commercial silicone**	6.7	4.9 *	73.1 *

* Sulfurization test conducted for 48 h.
